# Supplementary material for: Distinct initiating events underpin the immune and metabolic heterogeneity of KRAS-mutant lung adenocarcinoma
Source: Nat Commun. 2019 Sep 13;10:4190. doi: 10.1038/s41467-019-12164-y (PMC6744438; doi:10.1038/s41467-019-12164-y)
Supplement: Supplementary file 1 — Supplementary Information [file 41467_2019_12164_MOESM1_ESM.pdf]

Supplementary Information

Distinct initiating events underpin the immune and metabolic heterogeneity of *KRAS*-mutant lung adenocarcinoma

Best *et al.*

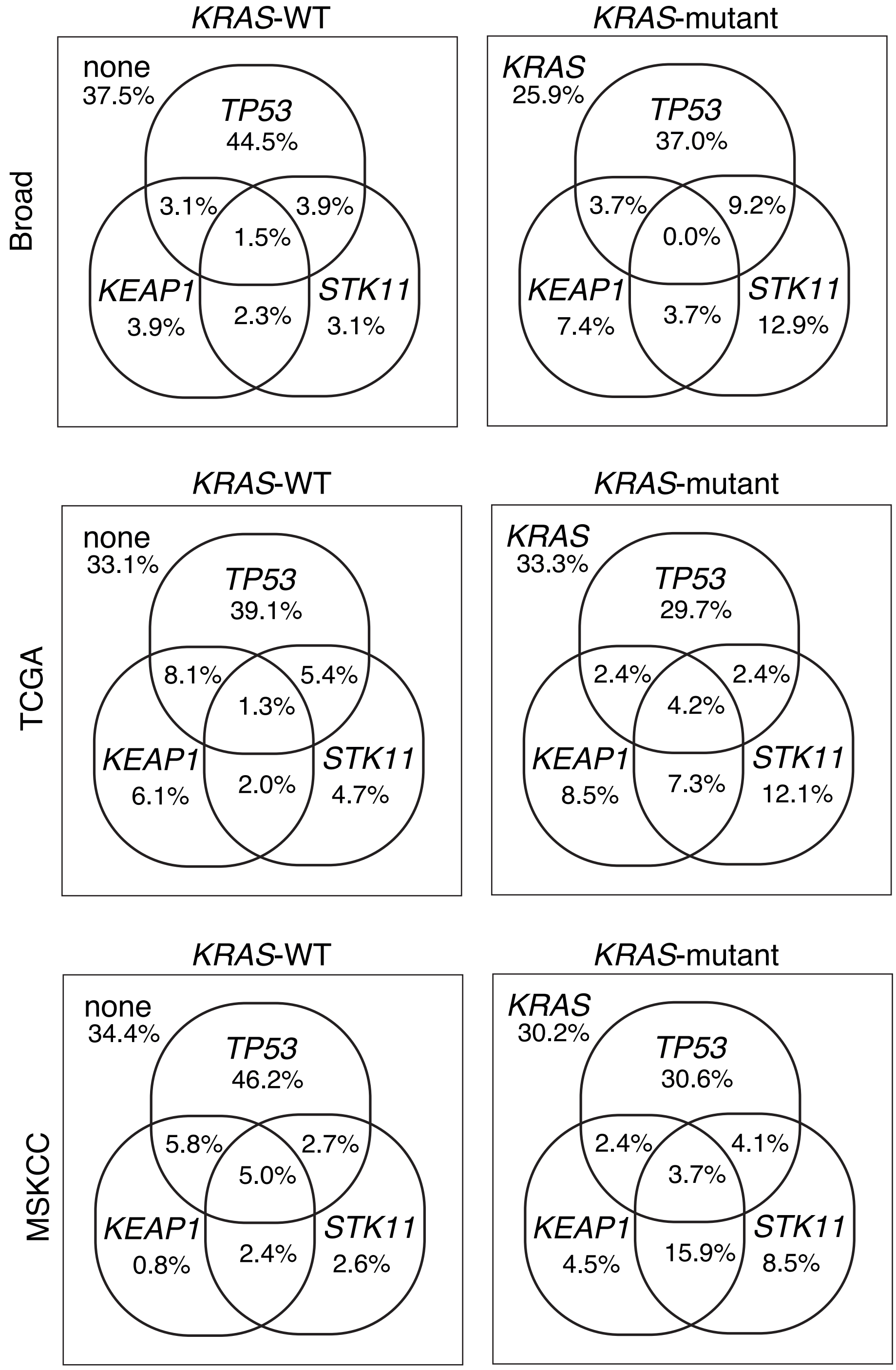

Supplementary Fig. 1 | Occurrence of *TP53*, *KEAP1* and *STK11* co-mutation in LUAD

Venn Diagram of co-mutation/mutual exclusivity of *KEAP1*, *TP53* and *STK11* mutations in *KRAS*-wild type (WT), left panels, or *KRAS*-mutant, right panels, in LUAD datasets from the Broad Institute, The Cancer Genome Atlas (TCGA) and Memorial Sloan Kettering Cancer Center (MSKCC). Related to Supplementary Table 1.

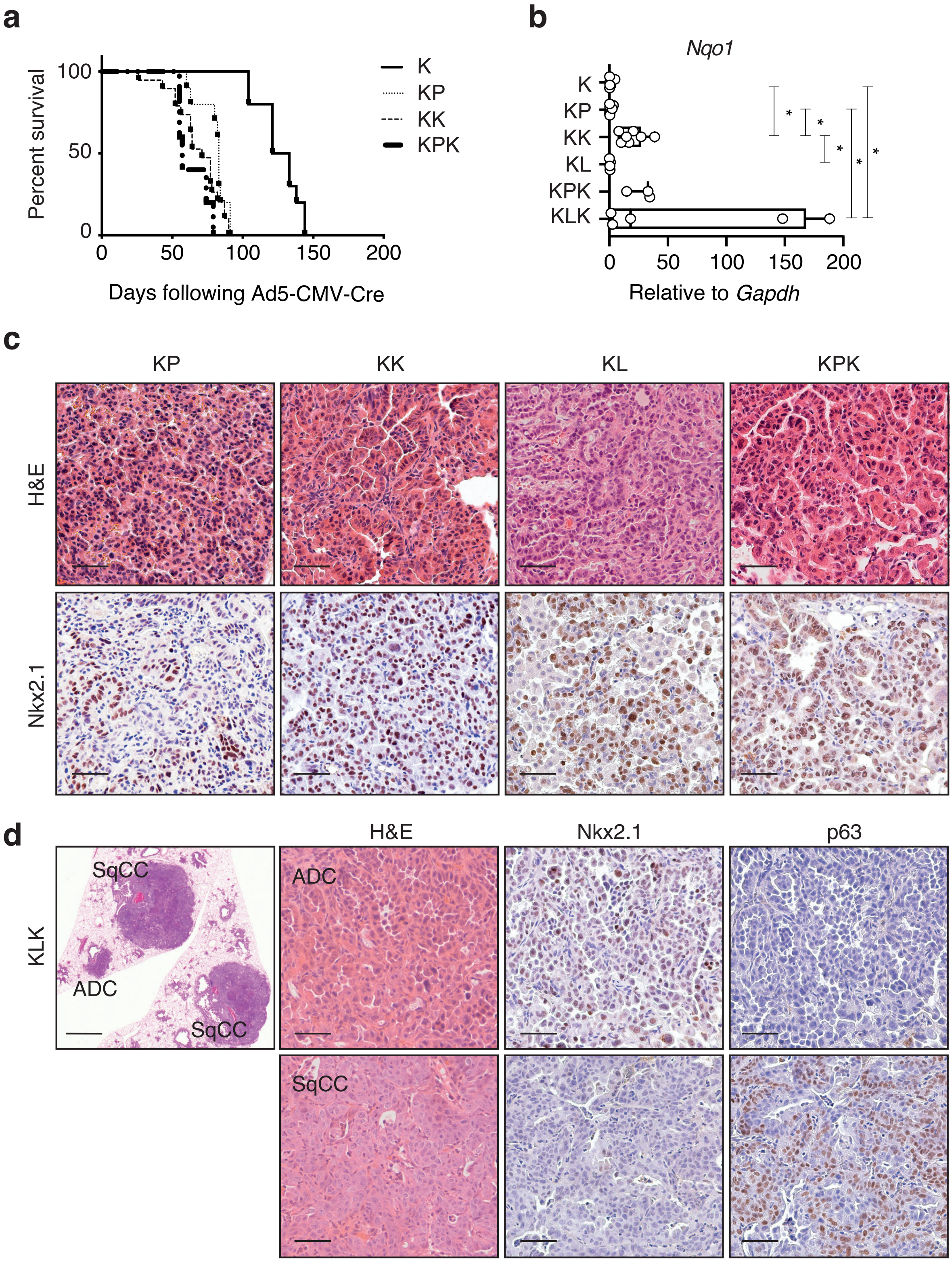

a
Percent survival
100
50
0
0
50
100
150
200
Days following Ad5-CMV-Cre
K
KP
KK
KPK
b
Nqo1
K
KP
KK
KL
KPK
KLK
*
*
*
*
*
0
50
100
150
200
Relative to Gapdh
c
KP
KK
KL
KPK
H&E
Nkx2.1
d
H&E
Nkx2.1
p63
KLK
SqCC
ADC
SqCC
ADC
SqCC

Supplementary Fig. 2 | Genetically engineered mouse models reflecting the heterogeneity of *KRAS*-mutant LUAD

(a) Kaplan-Meyer survival curve of K (n=10), KP (n=10), KK (n=19) and KPK (n=5) mice following intranasal inhalation of Ad5-CMV-Cre. Median survival and range provided in Table 1. **(b)** Expression of *Nqo1* in tumor pieces from K (n=4), KP (n=4), KK (n=7), KL (n=5), KPK (n=3) and KLK (n=5) lungs. Brown-Forsythe and Welch ANOVA test/Holm-Sidak's multiple comparisons test: K v KK: *p=0.0487; KK v KP *p=0.0487; KK v KL *p=0.0476; K v KLK *p=0.05; KP v KLK *p=0.05. Box plot min-max. (**c)** Representative hematoxylin and eosin (H&E) staining and Nkx2.1 (TTF-1) immunostaining of tumors from KP, KK, KL and KPK mice. Scale, 50 µm. (**d)** Representative H&E and Nkx2.1, p63 immunostaining of adenocarcinoma (ADC, above) and squamous cell carcinoma (SqCC, below) nodules in KLK lung tumors. Scale, 1 mm left panel; 50 µm right panels.

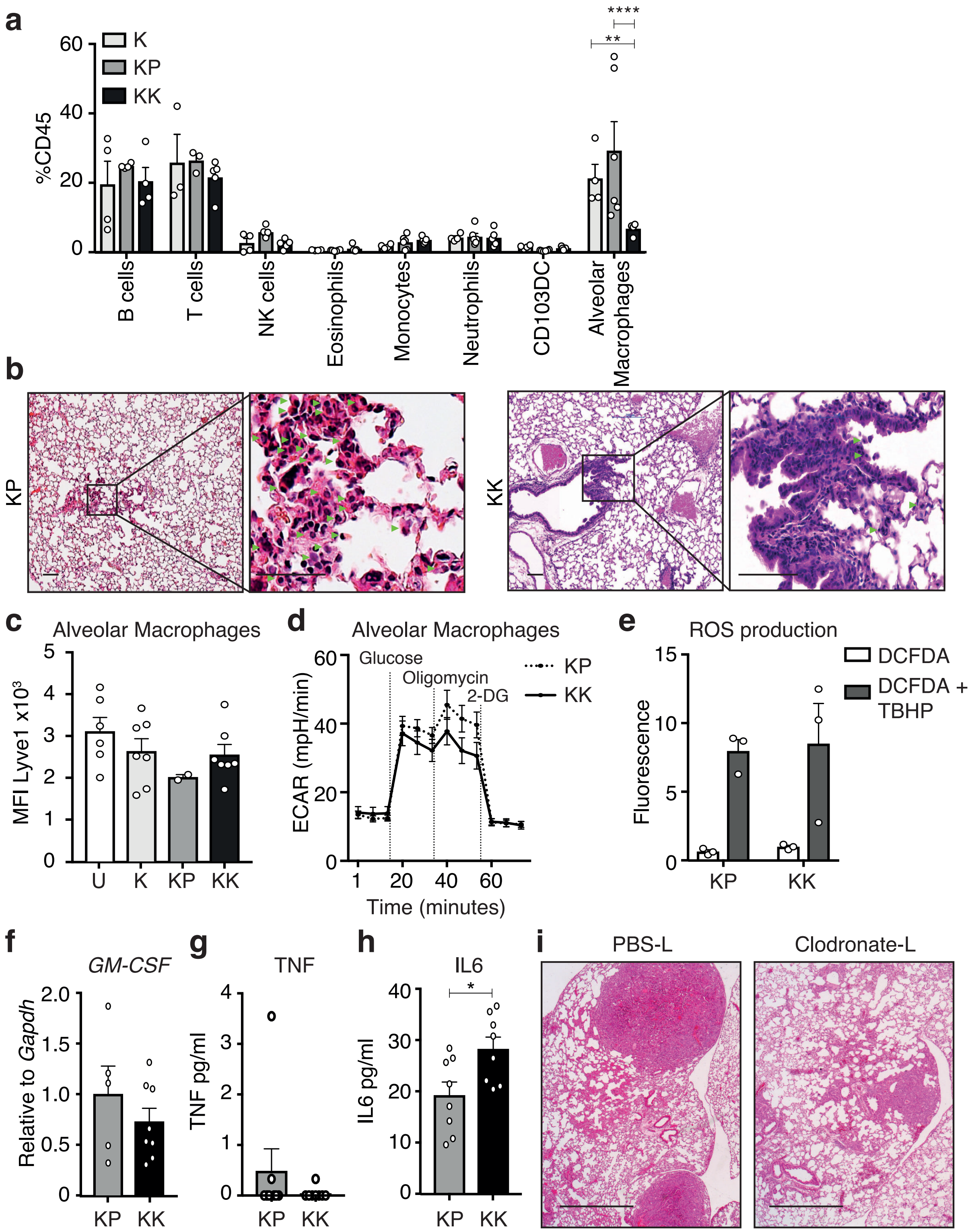

a
K
KP
KK
%CD45
B cells
T cells
NK cells
Eosinophils
Monocytes
Neutrophils
CD103DC
Alveolar Macrophages
b
KP
KK
c
Alveolar Macrophages
MFI Lyve1 x10³
U
K
KP
KK
d
Alveolar Macrophages
Glucose
Oligomycin
2-DG
KP
KK
ECAR (mpH/min)
Time (minutes)
e
ROS production
DCFDA
DCFDA + TBHP
Fluorescence
KP
KK
f
GM-CSF
Relative to Gapdh
KP
KK
g
TNF
TNF pg/ml
KP
KK
h
IL6
IL6 pg/ml
KP
KK
i
PBS-L
Clodronate-L

Supplementary Fig. 3 | Alveolar macrophage infiltrate are phenotypically similar in inflamed and non-inflamed models of LUAD

(a) Quantification of immune subsets (B cells: $CD3^-B220^+$; T cells: $CD3^+B220^-$; NK cells: $CD3^-Dx5^+Nkp46^+$; Eosinophils: $CD11c^-CD11b^+Ly6G^-SSC^{hi}$; Monocytes: $CD11c^-CD11b^+Ly6G^-SSC^{lo}$; Neutrophils: $CD11c^-CD11b^+Ly6G^+$; CD103DC: $CD11c^+CD11b^-CD103^+$; Alveolar macrophages: $CD11c^+CD11b^-CD103^-$) as a proportion of $CD45^+$ cells in the lungs of K (n=4), KP (n=6) and KK (n=5) mice. Two-way ANOVA/Tukey's multiple comparisons test, alveolar macrophages: K v KK **p=0.0061, KP v KK ****p<0.0001. Mean ± SEM. **(b)** Representative H&E of KP and KK lung sections 3 weeks following intranasal inhalation of Ad5-CMV-Cre. Magnification of hyperplastic region, with green arrows indicating alveolar macrophages. Scale, 100 µm. **(c)** Quantification of mean fluorescence intensity (MFI) of Lyve-1 expression on alveolar macrophages in uninfected (U; n=6) and K (n=7), KP (n=2) and KK (n=7) 3 months post Ad5-CMV-Cre administration. Mean ± SEM. **(d)** Glycolytic assay of extracellular acidification rate in KP (n=4) and KK (n=3) FACS isolated alveolar macrophages 3 months following Ad5-CMV-Cre infection. Mean ± SEM. **(e)** Quantification of ROS production in freshly isolated alveolar macrophages from KP (n=3) and KK (n=3) tumor-bearing lungs. Mean ± SEM. **(f)** Expression of *GM-CSF* in lung tumor pieces from KP (n=5) and KK (n=7). Expression relative to *Gapdh* housekeeper control and quantified relative to KP. Mean ± SEM. **(g)** ELIZA of TNF levels in KP (n=4) and KK (n=4) tumor cell line supernatant. Mean ± SEM. **(h)** ELIZA of IL6 levels in KP (n=4) and KK (n=4) tumor cell line supernatant. Unpaired students t test *p=0.02. Mean ± SEM. **(i)** Representative H&E of PBS- and Clodronate-liposome (L) treated mice. Scale, 1 mm.

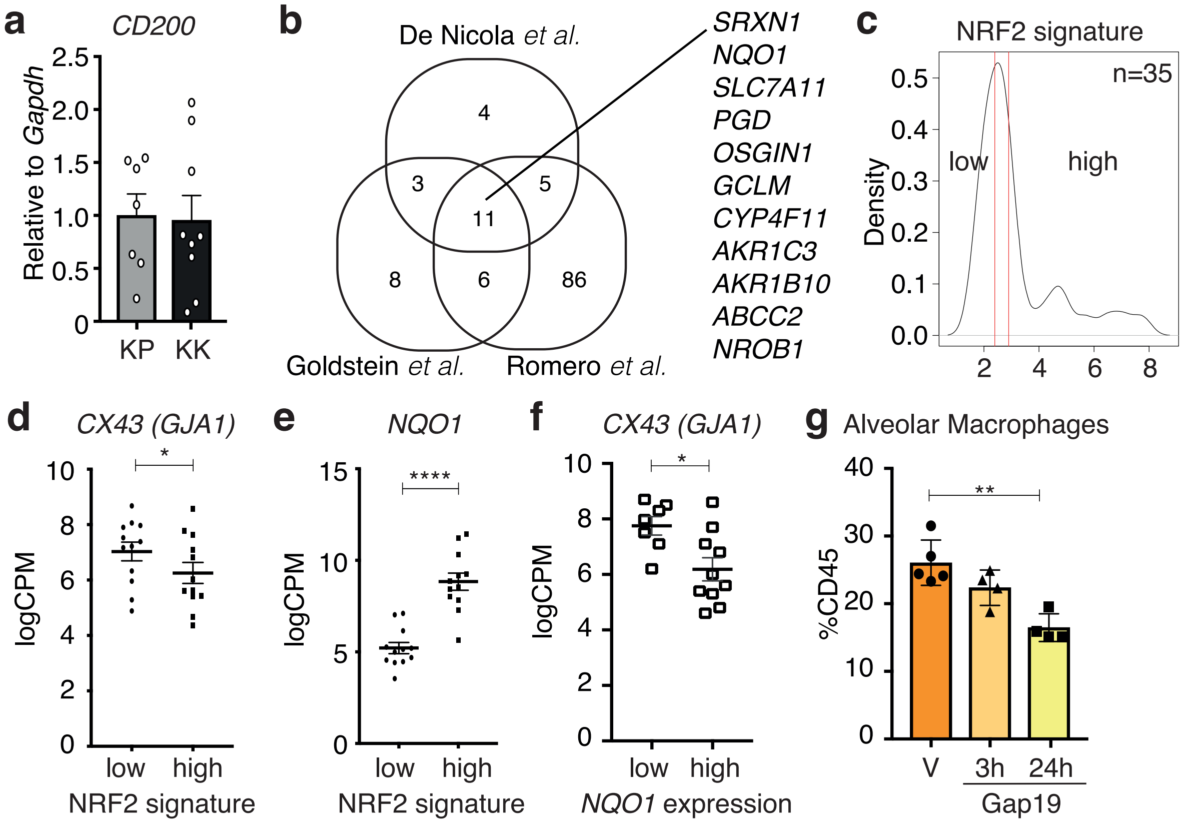

a
CD200
Relative to Gapdh
2.5
2.0
1.5
1.0
0.5
0
KP
KK
b
De Nicola et al.
Goldstein et al.
Romero et al.
4
3
5
11
8
6
86
SRXN1
NQO1
SLC7A11
PGD
OSGIN1
GCLM
CYP4F11
AKR1C3
AKR1B10
ABCC2
NROB1
c
NRF2 signature
n=35
low
high
Density
0.5
0.4
0.3
0.2
0.1
0.0
2
4
6
8
d
CX43 (GJA1)
*
logCPM
10
8
6
4
2
0
low
high
NRF2 signature
e
NQO1
****
logCPM
15
10
5
0
low
high
NRF2 signature
f
CX43 (GJA1)
*
logCPM
10
8
6
4
2
0
low
high
NQO1 expression
g
Alveolar Macrophages
**
%CD45
40
30
20
10
0
V
3h
24h
Gap19

Supplementary Fig. 4 | CX43 is a key mediator of the interaction between alveolar macrophages and lung epithelium

(a) Expression of *CD200* in lung tumor pieces from KP (n=5) and KK (n=7) mice. Expression relative to *Gapdh* housekeeper control and quantified relative to KP. Mean ± SEM. **(b)** Venn diagram comparing the published NRF2 gene signatures from De Nicola *et al.*[1], Romero *et al.*[2] and Goldstein *et al.*[3]. Eleven common genes highlighted are the NRF2 consensus signature. **(c)** Stratification of n=35 *KRAS*-mutant LUAD TCGA patient samples into low or high expression of the NRF2 signature. "Low" denotes lowest 33.33% samples, cutoff expression value 2.39; "High" denotes highest 66.66% samples, cutoff expression value 2.89. **(d)** Expression of *CX43/GJA1* in the low and high subsets of *KRAS*-mutant LUAD TCGA. FDR *p=0.01465. Mean ± SEM. **(e)** Expression of *NQO1* in the low and high subsets of *KRAS*-mutant LUAD TCGA. FDR ****p=1.114×10^{-8}. Mean ± SEM. **(f)** Analysis of *CX43/GJA1* expression in $NQO1^{low}$ (n=7) and $NQO1^{high}$ (n=10) *KRAS*-mutant LUAD TCGA patient samples. FDR *p=0.015. Mean ± SEM. **(g)** Quantification of alveolar macrophages in the lungs of C57Bl/6 mice treated with vehicle (V; n=5), 3 hours exposure to Gap19 (3h; n=4) and 24 hours exposure to Gap19 (24h; n=4). Unpaired t test V v 24h **p=0.0016. Mean ± SD.

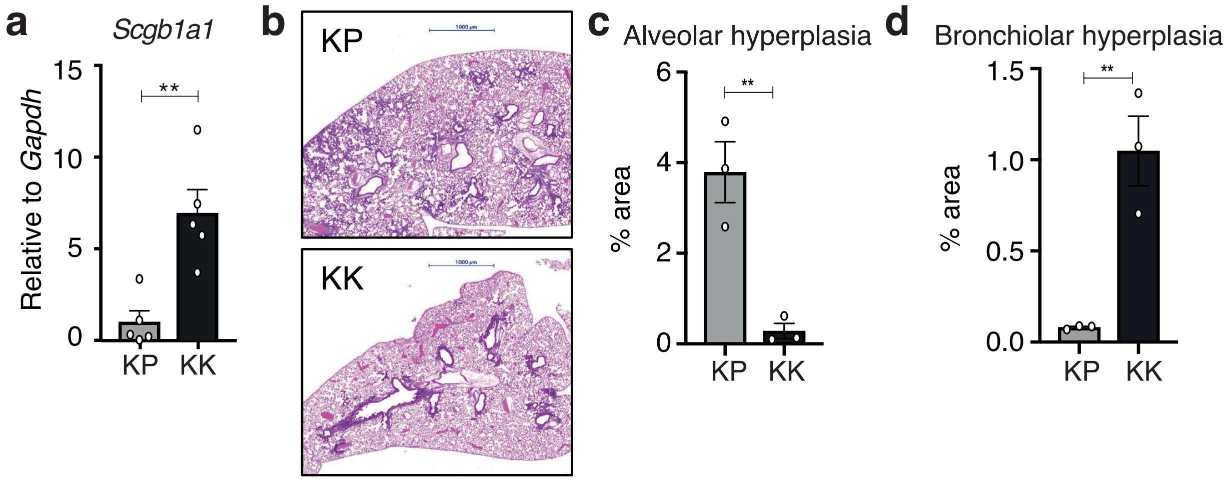

Supplementary Fig. 5 | *Keap1*-deficient *Kras*^{G12D}-induced tumors arise from a bronchiolar cell-of-origin

(a) Expression of *Scgb1a1* in lung tumor pieces from KP (n=5) and KK (n=5) mice. Expression relative to *Gapdh* housekeeper control and quantified relative to KP. Unpaired t test **p=0.0032. Mean ± SEM. **(b)** Representative low power H&E image of KP and KK lungs 3 weeks following Ad5-CMV-Cre. Scale, 1mm. **(c)** Quantification of alveolar hyperplasia in KP and KK mice (n=3/genotype) 3 weeks post infection with Ad5-CMV-Cre. Quantified as percentage area alveolar hyperplasia in total lung histology area. Unpaired t test **p=0.0072. Mean ± SEM. **(d)** Quantification of bronchiolar hyperplasia in KP and KK mice (n=3/genotype) 3 weeks post infection with Ad5-CMV-Cre. Quantified as percentage area bronchiolar hyperplasia in total lung histology area. Unpaired t test **p=0.0072. Mean ± SEM.

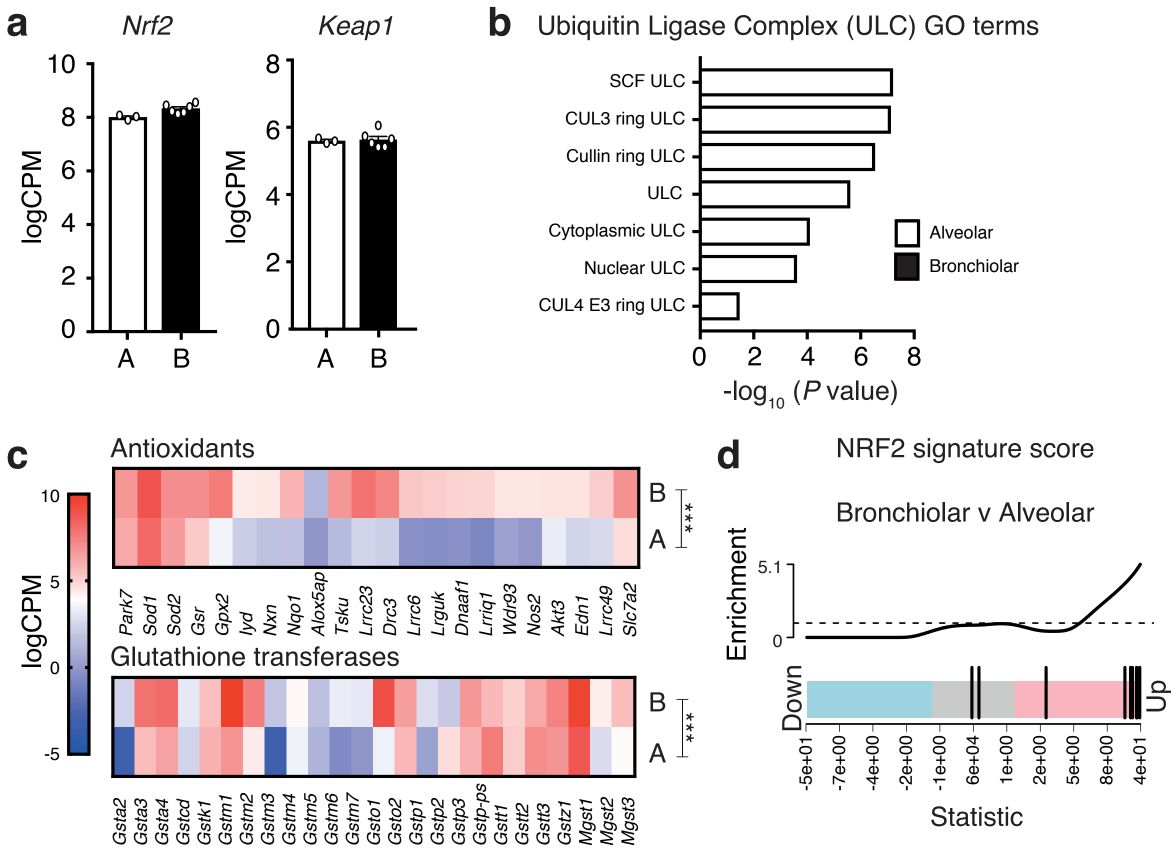

a
Nrf2
Keap1
logCPM
A
B
b
Ubiquitin Ligase Complex (ULC) GO terms
SCF ULC
CUL3 ring ULC
Cullin ring ULC
ULC
Cytoplasmic ULC
Nuclear ULC
CUL4 E3 ring ULC
Alveolar
Bronchiolar
-log10 (P value)
c
Antioxidants
Park7 Sod1 Sod2 Gsr Gpx2 Iyd Nxn Nqo1 Alox5ap Tsku Lrrc23 Drc3 Lrrc6 Lrguk Dnaaf1 Lrriq1 Wdr93 Nos2 Akt3 Edn1 Lrrc49 Slc7a2
Glutathione transferases
Gsta2 Gsta3 Gsta4 Gstcd Gstk1 Gstm1 Gstm2 Gstm3 Gstm4 Gstm5 Gstm6 Gstm7 Gsto1 Gsto2 Gstp1 Gstp2 Gstp3 Gstp-ps Gstt1 Gstt2 Gstt3 Gstz1 Mgst1 Mgst2 Mgst3
logCPM
***
d
NRF2 signature score
Bronchiolar v Alveolar
Enrichment
Down
Up
Statistic

Supplementary Fig. 6 | The Nrf2 pathway is highly active in bronchiolar cells

(a) Expression of *Nrf2* and *Keap1* in alveolar (A) and bronchiolar (B) cells (n=3). Quantified logCPM from RNA-sequencing data. Mean ± SEM. **(b)** Analysis of differentially expressed Gene Ontogeny (GO) terms from c5CC list upregulated in alveolar cells. ULC, ubiquitin ligase complex. Quantified by p-value significance. **(c)** Expression of antioxidant gene panel (above) and glutathione transferase gene panel (below) in bronchiolar (B) and alveolar (A) lung epithelial cells. Color intensity directly relates to RNA-sequencing expression as average logCPM. Differential ROAST analysis ***p=0.0005 up-regulation in B compared to A, in both antioxidant and glutathione transferase panels. **(d)** Barcode plot of the NRF2 signature score genes (black bars) applied the differentially expressed genes in bronchiolar v alveolar dataset. Enrichment of signature is graphed above and indicated by the line. Significance is generated using moderated t-statistics, computed by empirical Bayes moderation of linear model fit. The blue and red regions denote significance.

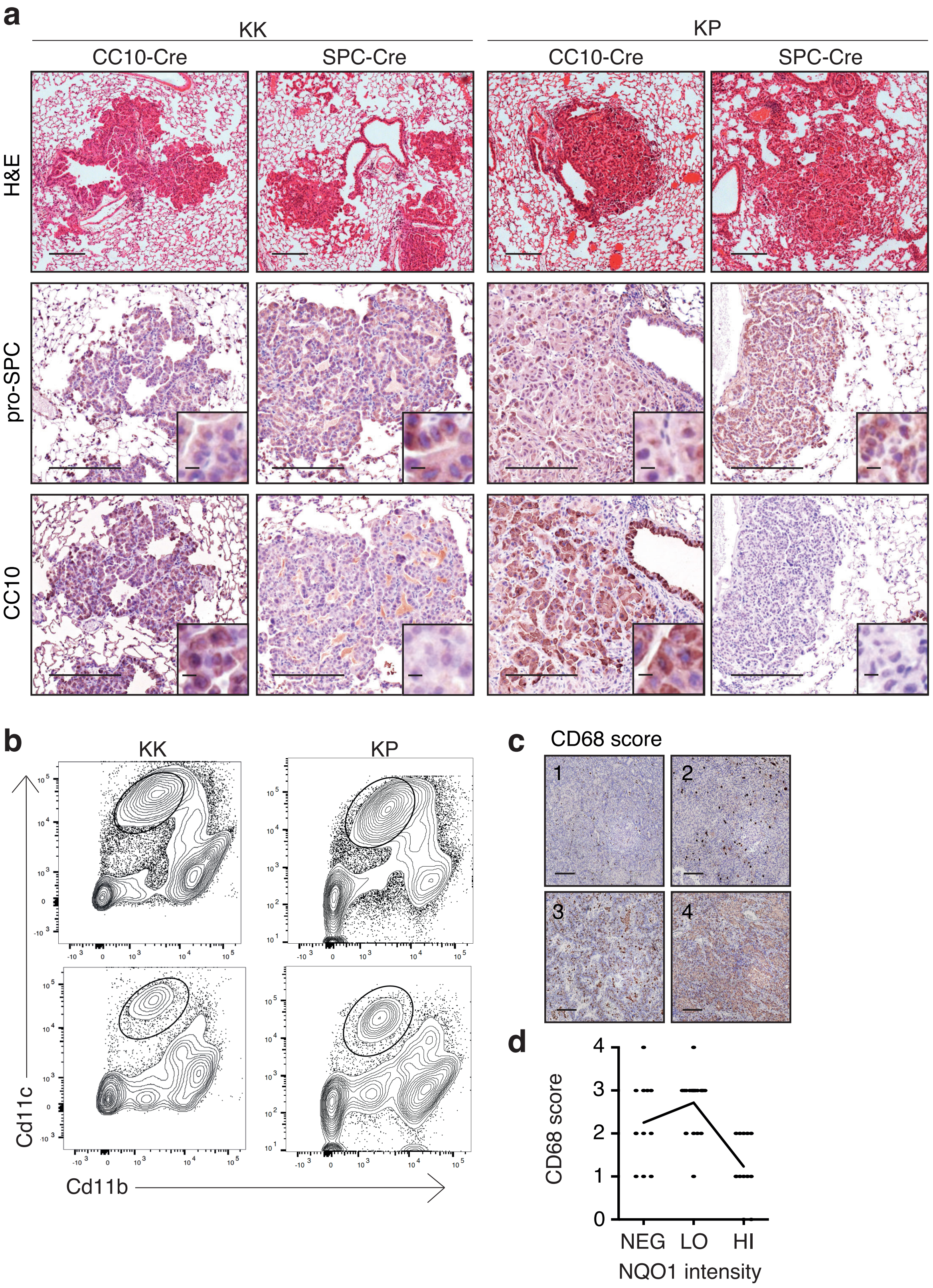

a
KK
KP
CC10-Cre
SPC-Cre
CC10-Cre
SPC-Cre
H&E
pro-SPC
CC10
b
KK
KP
SPC-Cre
CC10-Cre
Cd11c
Cd11b
c
CD68 score
1
2
3
4
d
CD68 score
NEG
LO
HI
NQO1 intensity

Supplementary Fig. 7 | Alveolar macrophage expansion is associated with an alveolar cell-of-origin

(a) Immunostaining panel of H&E, pro-SPC and CC10 in representative KK and KP lung tissue 3 months post Ad5-CC10-Cre (CC10-Cre) or Ad5-SPC-Cre (SPC-Cre) infection. Scale, 200 μm; Inset, 10 μm. **(b)** Representative flow cytometry plots of alveolar macrophage population ($CD103^{-}CD11c^{+}CD11b^{-}$) in KK and KP lung tissue 3 months following Ad5-CMV-Cre, Ad5-SPC-Cre or Ad5-CC10-Cre administration. **(c)** Representative immunostaining of CD68 in patient samples to indicate score intensity from 1 to 4. Scale, 200 μm. **(d)** Quantification of CD68 relative to NQO1 staining intensity in *KRAS*-mutant lung adenocarcinoma patient samples (n=47).

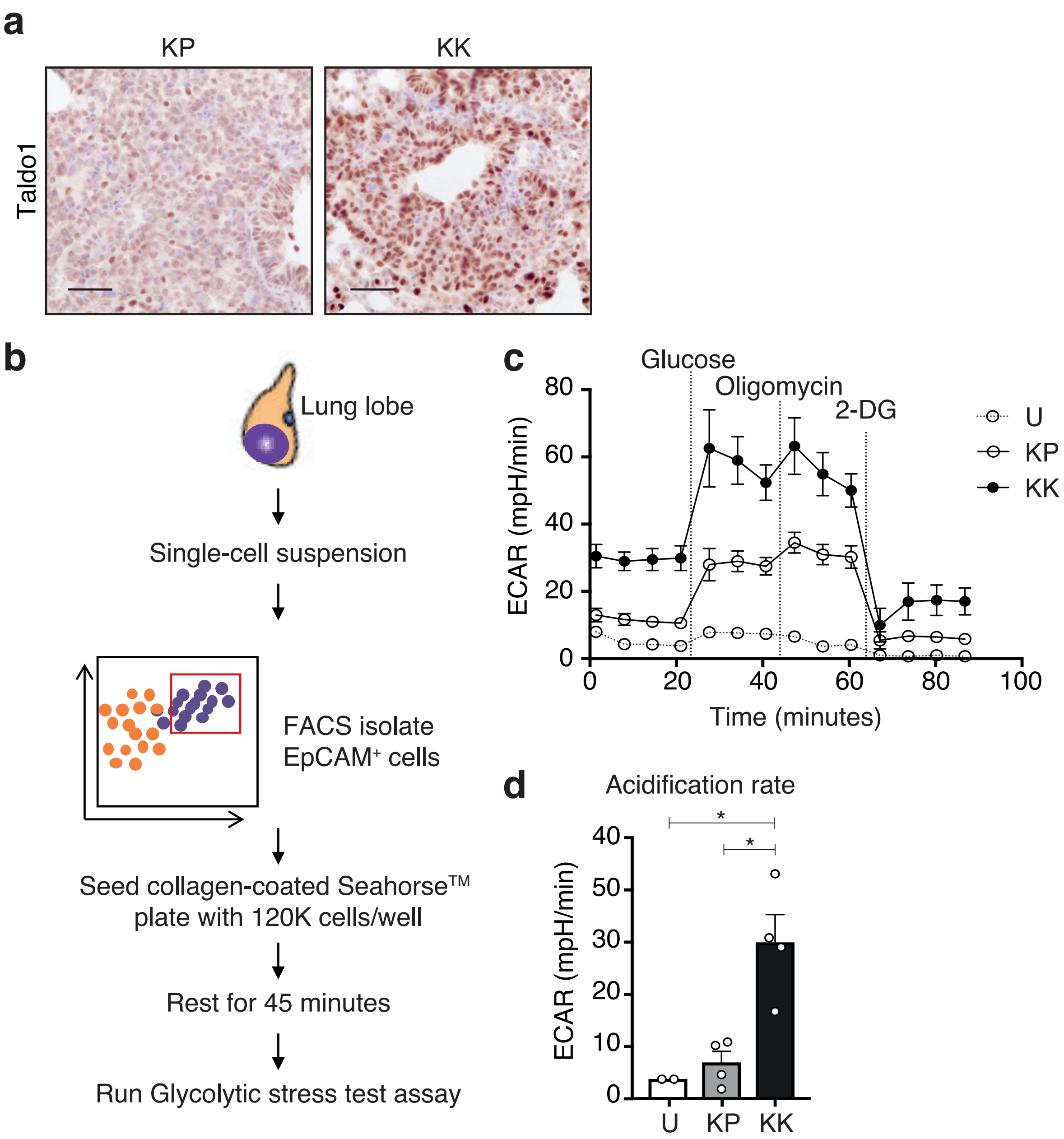
a
KP
KK
Taldo1
b
Lung lobe
Single-cell suspension
FACS isolate
EpCAM+ cells
Seed collagen-coated SeahorseTM
plate with 120K cells/well
Rest for 45 minutes
Run Glycolytic stress test assay
c
Glucose
Oligomycin
2-DG
U
KP
KK
ECAR (mpH/min)
0
20
40
60
80
100
Time (minutes)
d
Acidification rate
*
*
ECAR (mpH/min)
0
10
20
30
50
40
U
KP
KK

Supplementary Fig. 8 | *Kras/Keap1* tumors have a high extracellular acidification rate

(a) Immunostaining of Taldo1 protein in KP and KK lung tissue 3 months following Ad5-CMV-Cre infection. Scale, 50 μm. **(b)** Schematic of protocol generated to perform glycolytic stress test Seahorse^{TM} assay on primary lung epithelial cells. Briefly, single-cell suspensions were generated from lung lobes and EpCAM$^+$ cells isolated by fluorescence activated cell sorting (FACS). Following re-counting, 120,000 cells/well were seeded onto collagen-coated wells and rested for 45 minutes prior to the assay setup. **(c)** Representative example of glycolytic assay of uninfected (U; n=2 experiments, pooled lungs of n=6 mice per experiment) and KP (n=4) and KK (n=4) flow cytometry isolated tumor cells 3 months following Ad5-CMV-Cre infection. Mean ± SD. 2-DG, 2-deoxy-glucose. **(d)** Quantification of acidification rate in of uninfected (U; n=2 experiments, n=6 mice per experiment) and KP (n=4) and KK (n=4) primary lung EpCAM$^+$ cells 3 months following Ad5-CMV-Cre infection. Ordinary one-way ANOVA/Holm-Sidak's multiple comparisons test U v KK *p=0.0111, KP v KK *p=0.0111. Mean ± SEM.

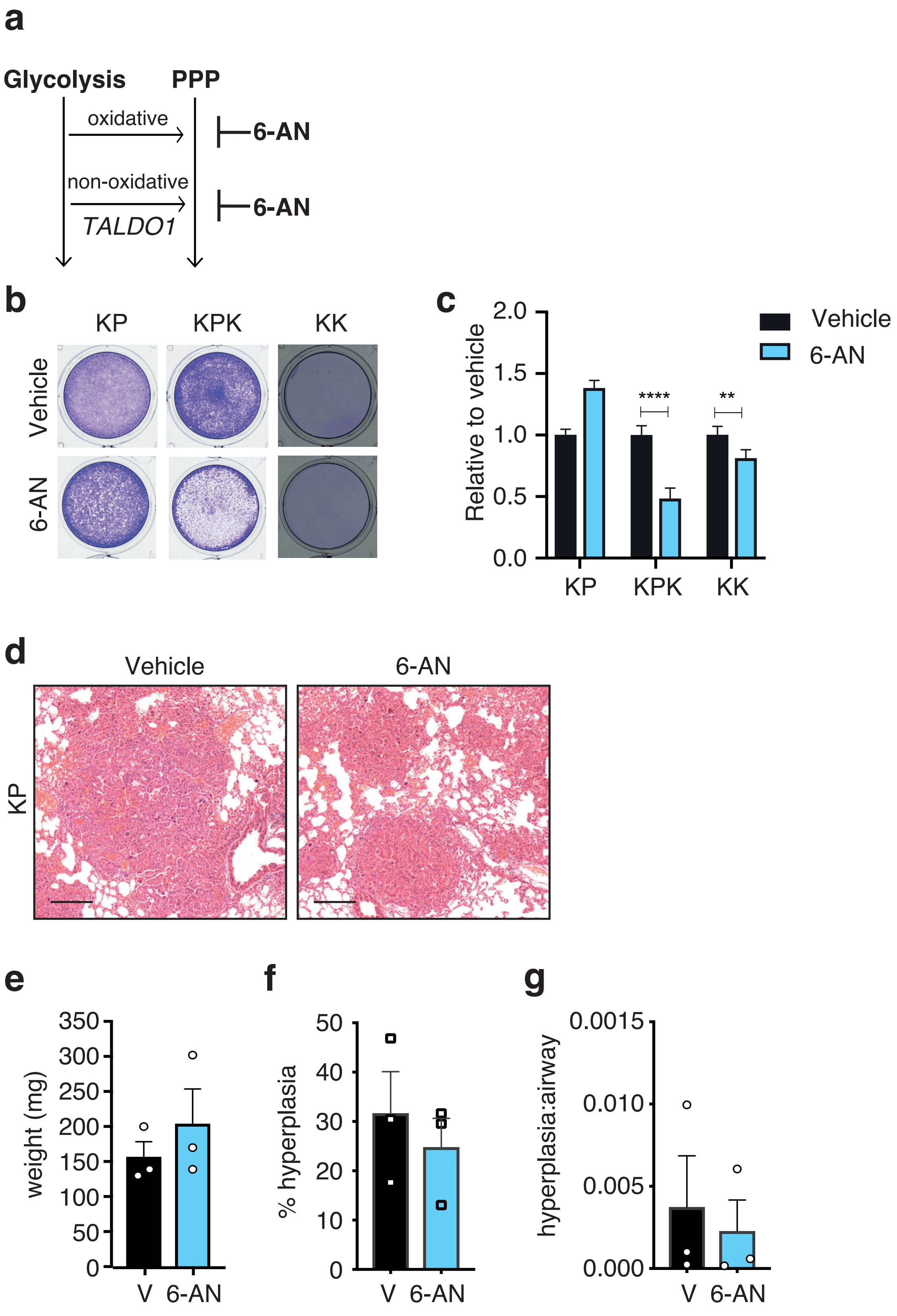

a
Glycolysis
PPP
oxidative
6-AN
non-oxidative
TALDO1
6-AN
b
KP
KPK
KK
Vehicle
6-AN
c
Relative to vehicle
2.0
1.5
1.0
0.5
0.0
Vehicle
6-AN
****
**
KP
KPK
KK
d
Vehicle
6-AN
KP
e
weight (mg)
350
300
250
200
150
100
50
0
V
6-AN
f
% hyperplasia
50
40
30
20
10
0
V
6-AN
g
hyperplasia:airway
0.0015
0.010
0.005
0.000
V
6-AN

Supplementary Fig. 9 | 6-AN inhibits the proliferation of *Keap1*-deficient tumors

(a) Schematic of 6-aminonicotinamide (6-AN) activity in blocking the oxidative and non-oxidative arms of the PPP. **(b)** Representative images of KP, KPK and KK primary mouse cell lines following 72 hours exposure to vehicle (DMSO) or 62.5 μM 6-AN colony assay. **(c)** Quantification of 6-AN colony assay from (b) relative to vehicle control. One biologically independent tumor cell line was used for each genotype, with each assay performed with 3 technical replicates over at least 3 independent experiments. Mean +/- SEM. **(d)** Representative H&E staining of lungs from KP mice treated with 6-AN or vehicle. Scale, 200 μm. **(e)** Left lobe lung weight of KP mice treated with 20 mg/kg 6-AN (n=3) or vehicle (n=3) for three cycles from 40 days post Ad5-CMV-Cre administration and collected at 64 days. Mean ± SEM. **(f)** Quantification of hyperplasia in H&E stained sections of 6-AN (n=3) or vehicle (n=3) treated mice. **(g)** Ratio of hyperplasia relative to large airway size in vehicle (n=3) and 6-AN (n=3) treated KP lungs.

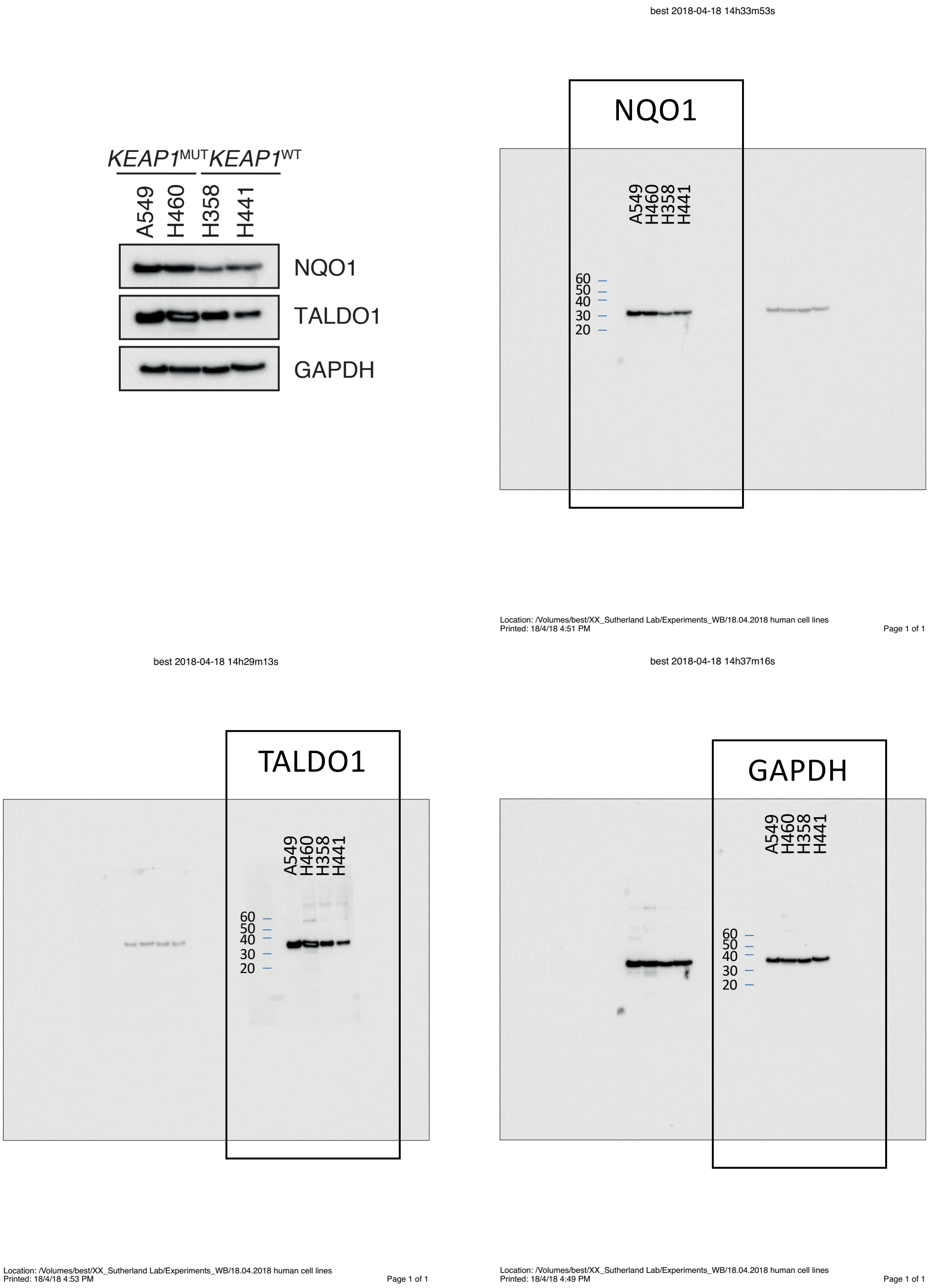

Supplementary Fig. 10 | Increased NQO1 and TALDO1 expression in *KRAS*-mutant NSCLC cell lines

Western blot of NQO1 and TALDO1 protein expression in *KRAS*^{MUT} NSCLC cell lines (*KEAP1*^{MUT}: A549, H460; *KEAP1*^{WT}: H358, H441). GAPDH provides the loading control. Uncropped images of Western blots with molecular weight marker indicated are provided.

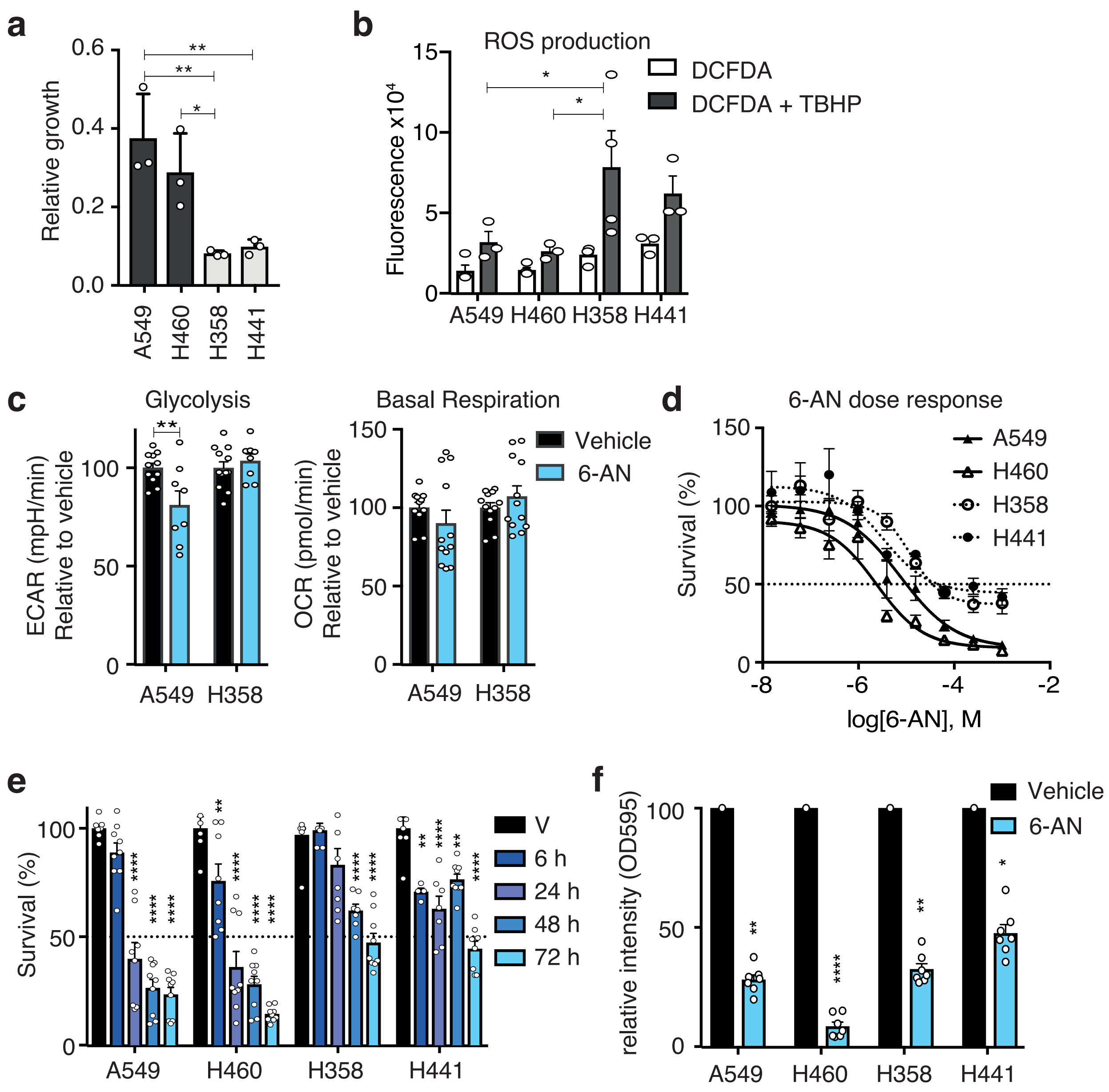

Supplementary Fig. 11 | *KEAP1*-mutant human NSCLC cell lines are sensitive to 6-AN

(a) Relative growth rates of *KRAS*^{MUT} NSCLC cell lines based on MTS assay absorbance. Ordinary one-way ANOVA/Tukey's multiple comparisons test, A549 v H358 **p=0.0067; A549 v H441 **p=0.0094; H460 v H358 *p=0.0428. n=3 per cell line, mean ± SD. **(b)** Quantification of ROS production in *KRAS*^{MUT} NSCLC cell lines. Two-way ANOVA/Sidak's multiple comparisons test, A549 v H358 *p=0.05, H460 v H358 *p=0.0262. n=3 independent experiments per cell line, mean ± SEM. **(c)** Glycolysis stress test assay of A549 and H358 cell lines exposed to vehicle (n=3) or 62.5 uM 6-AN (n=3) for 6 hours. Two-way ANOVA/Sidak's

multiple comparisons test, A549 Vehicle v 6-AN **p=0.0046. Mean ± SEM. Basal respiration assay of A549 and H358 cell lines exposed to vehicle (n=3 independent experiments per cell line) or 62.5 uM 6-AN (n=3 independent experiments per cell line) for 6 hours. Mean ± SEM. **(d)** Dose survival curve of 6-AN treatment in *KRAS*^{MUT} NSCLC cell lines. n=3 independent experiments per cell line, IC_{50}: 11.48 µM, 2.45 µM, 38.9 µM and 50.12 µM, respectively. Mean ± SEM. **(e)** Survival of *KRAS*^{MUT} NSCLC cell lines treated with 62.5 µM 6-AN for 6, 24, 48 or 72 hours (n=3 independent experiments per cell line) relative to vehicle within each cell line. Two-way ANOVA/Dunnett's multiple comparisons test, A549 ****p<0.0001; H460 V v 6h **p=0.0045, V v 24, 48, 72h ****p<0.0001; H358 V v 48, 72h ****p<0.0001; H441 V v 6h **p=0.0031, V v 24, 72h ****p<0.0001, V v 48h **p=0.0029. Mean ± SEM. **(f)** Quantification of crystal violet stain in colony assay from *KRAS*^{MUT} NSCLC cell lines treated with 62.5 µM 6-AN for 72 hours. Quantified relative to the vehicle of each cell line (n=7 independent experiments per cell line). Two-way ANOVA/Sidak's multiple comparisons test vehicle v 6-AN, A549 **p=0.0024; H460 ****p<0.0001; H358 **p=0.0038; H441 *p=0.0239. Mean ± SEM.

Supplementary Table 1: *KRAS* and co-occurring mutation frequencies in LUAD datasets

***KRAS* status**	**Co-mutation**	**MSKCC[a]**	**TCGA[b]**	**Broad[c]**	**CLCGP[d]**	**MSKCC[e]**	**TOTAL**	**%**
WT	*KEAP1*	5	9	5	N/A	0	**19**	2.1
	TP53	286	58	57		9	**410**	44.5
	STK11	16	7	4		2	**29**	3.1
	KEAP1+TP53	36	12	4		2	**54**	5.9
	KEAP1+STK11	15	3	3		1	**22**	2.4
	KEAP1+TP53+STK11	31	2	2		0	**35**	3.8
	TP53+STK11	17	8	5		1	**31**	3.4
	None	213	49	48		11	**321**	34.9
	**TOTAL**	**619**	**148**	**128**		**26**	**921**	
MUT	*KEAP1*	11	14	4	11	0	**40**	6.4
	TP53	75	49	20	38	3	**185**	29.5
	STK11	21	20	7	19	0	**67**	10.7
	KEAP1+TP53	6	4	2	1	1	**14**	2.2
	KEAP1+STK11	39	12	2	7	1	**61**	9.7
	KEAP1+TP53+STK11	9	7	0	1	0	**17**	2.7
	TP53+STK11	10	4	5	6	0	**25**	4
	None	74	55	14	72	3	**218**	34.8
	**TOTAL**	**245**	**165**	**54**	**155**	**8**	**627**	

[a]Jordan *et al.*[4]
[b]The Cancer Genome Atlas Research Network (TCGA)[5]
[c]Imielinski *et al.*[6]
[d]Clinical Lung Cancer Genome Project (CLCGP)[7]
[e]Rizvi *et al.*[8]

Supplementary Table 2: Primer List

Primer	Sequence	Source
Genotyping *Keap1*	5'-CGAGGAAGCGTTTGCTTTAC-3', 5'-GAGTCACCGTAAGCCTGGTC-3'	Blake *et al.*[9]
Genotyping *Kras*	5'-CGCAGACTGTAGAGCAGCG-3', 5'-CCATGGCTTGAGTAAGTCTGC-3'	Jackson *et al.*[10]
Genotyping *p53*	5'-AAGGGGTATGAGGGACAAGG-3', 5'-GAAGACAGAAAAGGGGAGGG-3'	Jonkers *et al.*[11]
Genotyping *Lkb1*	5'-ATCGGAATGTGATCCAGCTT-3', 5'-ACGTAGGCTGTGCAACCTCT-3'	Bardeesy *et al.*[12]
Genotyping *EYFP*	5'-AAGACCGCGAAGAGTTTGTC-3', 5'-AAAGTCGCTCTGAGTTGTTAT-3', 5'-GGAGCGGGAGAAATGGATATG-3'	Srinivas *et al.*[13]; Soriano[14]
Quantitative RT-PCR *Nqo1*	5'-CGACAACGGTCCTTTCCAGA-3', 5'-GCAGGATGCCACTCTGAATC-3'	Best *et al.*[15]
Quantitative RT-PCR *Cx43*	5'-GAACACGGCAAGGTGAAGAT-3', 5'-GAGCGAGAGACACCAAGGAC-3'	This paper
Quantitative RT-PCR *CD200*	5'-AGTGGTGACCCAGGATGAA-3', 5'-TACTATGGGCTGTACATAG-3'	This paper
Quantitative RT-PCR *Scgb1a1*	5'-ATCGCCATCACAATCACTACTG-3', 5'-CAGTCTCTTCAGCTGGGTGC-3'	Rock *et al.*[16]
Quantitative RT-PCR *Sftpc*	5'-CACTGGCCTCGTGGTGTATG-3', 5'-CCTGCAGAGAGCATTCCATC-3'	Kajstura *et al.*[17]
Quantitative RT-PCR *G6PD*	5'-GTCCAGAATCTCATGGTGCTGA-3', 5'-GCAATGTTGTCTCGATTCCAGA-3'	Best *et al.*[15]
Quantitative RT-PCR *Tkt*	5'-CGAAACCCTCACAATGATCG-3', 5'-TTCCTCAGGTTCAGCAGCTC-3'	Best *et al.*[15]
Quantitative RT-PCR *Pgd*	5'-ATGCCAGGAGGGAACAAAG-3', 5'-GTTCTCCGGTTCCCACTTTT-3'	Mitsuishi *et al.*[18]
Quantitative RT-PCR *Taldo1*	5'-TTATCATCAACCTGGGAGGG-3', 5'-GCGAAGGAGAAAAGCAGTGT-3'	Best *et al.*[15]
Quantitative RT-PCR *Me1*	5'-GGAGCTCCAGGTCCTTAGAA-3', 5'-TGAGCACGCTGTAGAAGAGC-3'	Mitsuishi *et al.*[18]
Quantitative RT-PCR *Gapdh*	5'-GCCAAGGTCATCCATGACAACT-3', 5'-GAGGGGCCATCCACAGTCTT-3'	Best *et al.*[15]

Supplementary Table 3: Antibody List

Antibody	Source	Catalogue	RRID	Dilution
Nqo1	Abcam	#ab34173	AB_2251526	1/1000
Nkx2.1/TTF-1	Dako	#M3575		1/200
P63	Biocare Medical	#CM163A	AB_10582730	1/100
Hmga2	Biocheck	#59170AP	AB_2616589	1/1000
F4/80	Hybridoma	N/A		1/100
CD68	Dako	M0876	AB_2074844	1/200
Pro-SPC	Merck	AB3786		1/2000
CC10	Merck	ABS1673		1/100
TALDO1	Sigma-Aldrich	#HPA048089	AB_2680260	1/200
GAPDH	Sigma-Aldrich	G8795	AB_1078991	1/1000
EpCAM	Biolegend	#118217	AB_1501158	1/200
CD104	Biolegend	#123603	AB_961034	1/200
CD45	Biolegend	#103114	AB_312979	1/250
CD31	Biolegend	#102417	AB_830756	1/250
CD3	Biolegend	#100305	AB_312670	1/400
CD19	Biolegend	#115511	AB_313646	1/400
CD4	Biolegend	#100536	AB_493701	1/400
CD8	Biolegend	#100708	AB_312747	1/400
FoxP3	Biolegend	#320013	AB_439749	1/200
DX5/CD49b	Biolegend	#108909	AB_313416	1/200
NKp46/CD335	Biolegend	#137603	AB_10552741	1/200
CD11c	Biolegend	#117309	AB_313778	1/200
CD11b	Biolegend	#101205	AB_312788	1/200
Ly6G	Biolegend	#127603	AB_1186105	1/400
CD103	Biolegend	#121405	AB_535948	1/400
Lyve1	Thermo Fisher	PA5-22783	AB_11152044	1/200

Supplementary Table 4: Software List

Resource	Source	Website
GraphPad Prism 7	GraphPad Software	http://www.graphpad.com/scientific-software/prism/
Zen	Zeiss	https://www.zeiss.com/microscopy/int/products/microscope-software/zen.html
FlowJo	FlowJo LLC	https://www.flowjo.com/solutions/flowjo
Image J	Softonic	https://imagej.net/Welcome
cBioPortal for Cancer Genomics	Cerami *et al.*[19]; Gao *et al.*[20]	http://www.cbioportal.org/
RTCGAToolbox R package	Samur[21]	https://bioconductor.org/packages/RTCGAToolbox
EGSEA R package	Alhamdoosh *et al.*[22]	https://bioconductor.org/packages/EGSEA
edgeR R package	Robinson *et al.*[23]	http://bioconductor.org/packages/edgeR
limma R package	Law *et al.*[24]; Ritchie *et al.*[25]	www.bioconductor.org/packages/limma
pheatmap R package	Kolde[26]	https://CRAN.R-project.org/package=pheatmap
R	R Core Team[27]	https://www.R-project.org/

Supplementary References

1. DeNicola GM*, et al.* NRF2 regulates serine biosynthesis in non-small cell lung cancer. *Nat Genet* **47**, 1475-1481 (2015).

2. Romero R*, et al.* Keap1 loss promotes Kras-driven lung cancer and results in dependence on glutaminolysis. *Nat Med* **23**, 1362-1368 (2017).

3. Goldstein LD*, et al.* Recurrent Loss of NFE2L2 Exon 2 Is a Mechanism for Nrf2 Pathway Activation in Human Cancers. *Cell Rep* **16**, 2605-2617 (2016).

4. Jordan EJ*, et al.* Prospective Comprehensive Molecular Characterization of Lung Adenocarcinomas for Efficient Patient Matching to Approved and Emerging Therapies. *Cancer Discov* **7**, 596-609 (2017).

5. Cancer Genome Atlas Research N. Comprehensive molecular profiling of lung adenocarcinoma. *Nature* **511**, 543-550 (2014).

6. Imielinski M*, et al.* Mapping the hallmarks of lung adenocarcinoma with massively parallel sequencing. *Cell* **150**, 1107-1120 (2012).

7. Clinical Lung Cancer Genome P, Network Genomic M. A genomics-based classification of human lung tumors. *Sci Transl Med* **5**, 209ra153 (2013).

8. Rizvi NA*, et al.* Cancer immunology. Mutational landscape determines sensitivity to PD-1 blockade in non-small cell lung cancer. *Science* **348**, 124-128 (2015).

9. Blake DJ*, et al.* Deletion of Keap1 in the lung attenuates acute cigarette smoke-induced oxidative stress and inflammation. *Am J Respir Cell Mol Biol* **42**, 524-536 (2010).

10. Jackson EL*, et al.* Analysis of lung tumor initiation and progression using conditional expression of oncogenic K-ras. *Genes Dev* **15**, 3243-3248 (2001).

11. Jonkers J, Meuwissen R, van der Gulden H, Peterse H, van der Valk M, Berns A. Synergistic tumor suppressor activity of BRCA2 and p53 in a conditional mouse model for breast cancer. *Nat Genet* **29**, 418-425 (2001).

12. Bardeesy N*, et al.* Loss of the Lkb1 tumour suppressor provokes intestinal polyposis but resistance to transformation. *Nature* **419**, 162-167 (2002).

13. Srinivas S*, et al.* Cre reporter strains produced by targeted insertion of EYFP and ECFP into the ROSA26 locus. *BMC Dev Biol* **1**, 4 (2001).

14. Soriano P. Generalized lacZ expression with the ROSA26 Cre reporter strain. *Nat Genet* **21**, 70-71 (1999).

15. Best SA*, et al.* Synergy between the KEAP1/NRF2 and PI3K Pathways Drives Non-Small-Cell Lung Cancer with an Altered Immune Microenvironment. *Cell Metab* **27**, 935-943 e934 (2018).

16. Rock JR, Gao X, Xue Y, Randell SH, Kong YY, Hogan BL. Notch-dependent differentiation of adult airway basal stem cells. *Cell Stem Cell* **8**, 639-648 (2011).

17. Kajstura J*, et al.* Evidence for human lung stem cells. *N Engl J Med* **364**, 1795-1806 (2011).

18. Mitsuishi Y*, et al.* Nrf2 redirects glucose and glutamine into anabolic pathways in metabolic reprogramming. *Cancer Cell* **22**, 66-79 (2012).

19. Cerami E*, et al.* The cBio cancer genomics portal: an open platform for exploring multidimensional cancer genomics data. *Cancer Discov* **2**, 401-404 (2012).

20. Gao J*, et al.* Integrative analysis of complex cancer genomics and clinical profiles using the cBioPortal. *Sci Signal* **6**, pl1 (2013).

21. Samur MK. RTCGAToolbox: a new tool for exporting TCGA Firehose data. *PLoS One* **9**, e106397 (2014).

22. Alhamdoosh M*, et al.* Combining multiple tools outperforms individual methods in gene set enrichment analyses. *Bioinformatics* **33**, 414-424 (2017).

23. Robinson MD, McCarthy DJ, Smyth GK. edgeR: a Bioconductor package for differential expression analysis of digital gene expression data. *Bioinformatics* **26**, 139-140 (2010).

24. Law CW, Chen Y, Shi W, Smyth GK. voom: Precision weights unlock linear model analysis tools for RNA-seq read counts. *Genome Biol* **15**, R29 (2014).

25. Ritchie ME*, et al.* limma powers differential expression analyses for RNA-sequencing and microarray studies. *Nucleic Acids Res* **43**, e47 (2015).

26. Kolde R. pheatmap: Pretty Heatmaps. (ed^(eds). R package version 1.0.12 edn (2019).

27. Team RC. R: A Language and Environment for Statistical Computing. (ed^(eds). version 3.5.3 edn (2019).
