# Supplementary material for: Distinct initiating events underpin the immune and metabolic heterogeneity of KRAS-mutant lung adenocarcinoma
Source: Nat Commun. 2019 Sep 13;10:4190. doi: 10.1038/s41467-019-12164-y (PMC6744438; doi:10.1038/s41467-019-12164-y)
Supplement: Supplementary file 3 — Description of Additional Supplementary Files [file 41467_2019_12164_MOESM3_ESM.pdf]

Description of Additional Supplementary Files

File Name: Supplementary Data 1
Description: outlines patient information and mutational status from the CLCGP cohort

File Name: Supplementary Data 2
Description: outlines the mutational status and immunophenotyping results of the St Vincent's KRAS-mutant patient cohort.
