# Supplementary material for: Distinct initiating events underpin the immune and metabolic heterogeneity of KRAS-mutant lung adenocarcinoma
Source: Nat Commun. 2019 Sep 13;10:4190. doi: 10.1038/s41467-019-12164-y (PMC6744438; doi:10.1038/s41467-019-12164-y)
Supplement: Supplementary file 6 — Reporting Summary [file 41467_2019_12164_MOESM6_ESM.pdf]

nature research

Corresponding author(s): *Double-blind peer review submissions: write DBPR and your manuscript number here instead of author names.*

Last updated by author(s): Jul 30, 2019

Reporting Summary

Nature Research wishes to improve the reproducibility of the work that we publish. This form provides structure for consistency and transparency in reporting. For further information on Nature Research policies, see Authors & Referees and the Editorial Policy Checklist.

Statistics

For all statistical analyses, confirm that the following items are present in the figure legend, table legend, main text, or Methods section.

n/a	Confirmed	
☐	☒	The exact sample size (n) for each experimental group/condition, given as a discrete number and unit of measurement
☒	☐	A statement on whether measurements were taken from distinct samples or whether the same sample was measured repeatedly
☐	☒	The statistical test(s) used AND whether they are one- or two-sided<br>*Only common tests should be described solely by name; describe more complex techniques in the Methods section.*
☒	☐	A description of all covariates tested
☒	☐	A description of any assumptions or corrections, such as tests of normality and adjustment for multiple comparisons
☐	☒	A full description of the statistical parameters including central tendency (e.g. means) or other basic estimates (e.g. regression coefficient) AND variation (e.g. standard deviation) or associated estimates of uncertainty (e.g. confidence intervals)
☐	☒	For null hypothesis testing, the test statistic (e.g. F, t, r) with confidence intervals, effect sizes, degrees of freedom and P value noted<br>*Give P values as exact values whenever suitable.*
☒	☐	For Bayesian analysis, information on the choice of priors and Markov chain Monte Carlo settings
☒	☐	For hierarchical and complex designs, identification of the appropriate level for tests and full reporting of outcomes
☒	☐	Estimates of effect sizes (e.g. Cohen's d, Pearson's r), indicating how they were calculated

Our web collection on statistics for biologists contains articles on many of the points above.

Software and code

Policy information about availability of computer code

Data collection	No software was used.
Data analysis	RNA-seq data analysis was performed in R (v 3.5.2) using the Bioconductor software: edgeR (v 3.24.3); limma (v 3.38.3); EGSEA (v 1.10.1); pheatmap (v 1.0.12) and RTGCAToolbox (v 2.12.1). Other statistical analysis was performed using Prism (GraphPad Software).

For manuscripts utilizing custom algorithms or software that are central to the research but not yet described in published literature, software must be made available to editors/reviewers. We strongly encourage code deposition in a community repository (e.g. GitHub). See the Nature Research guidelines for submitting code & software for further information.

Data

Policy information about availability of data

All manuscripts must include a data availability statement. This statement should provide the following information, where applicable:

- Accession codes, unique identifiers, or web links for publicly available datasets
- A list of figures that have associated raw data
- A description of any restrictions on data availability

The data that support the findings of this study are available from the corresponding author upon reasonable request.

Field-specific reporting

Please select the one below that is the best fit for your research. If you are not sure, read the appropriate sections before making your selection.

☒ Life sciences ☐ Behavioural & social sciences ☐ Ecological, evolutionary & environmental sciences

Life sciences study design

All studies must disclose on these points even when the disclosure is negative.

Sample size	All animal experiments involved n=2-10 mice per genotype per group.
Data exclusions	Exclusion criteria was established at the beginning of the project whereby mice that had not been successfully infected intranasally with Adenovirus-Cre were excluded from experimental groups. Unsuccessful infection was determined by (1) the weight of the superior lobe was that of an uninfected littermate control (up to 100mg) AND (2) the histology of the lung lobes had no indication of abnormalities.
Replication	All experiments were performed on greater than one occasion (cell line experiments were performed on at least 3 occasions), with replicates included on the same graph for the final data representation. The definition of occasion in the context of mouse experiments is: different day, different mouse litters AND different flow cytometry data acquisition
Randomization	All mice were randomized into experiments, both for Adenovirus-Cre infection and for treatment studies, by researchers blinded to mouse condition (i.e. only the animal technician was privy to relative health (indicative of tumor burden) of mouse prior to treatment study allocation).
Blinding	Blinding was applied for all histological interpretation, and mouse treatment study allocation (described above). The application of blinding for mouse FACS experiments and cell line experiments was not possible due to the knowledge of the mouse number and allocation, and the name of the cell line being used in experiments.

Reporting for specific materials, systems and methods

We require information from authors about some types of materials, experimental systems and methods used in many studies. Here, indicate whether each material, system or method listed is relevant to your study. If you are not sure if a list item applies to your research, read the appropriate section before selecting a response.

Materials & experimental systems

n/a	Involved in the study	
☐	☒	Antibodies
☐	☒	Eukaryotic cell lines
☒	☐	Palaeontology
☐	☒	Animals and other organisms
☐	☒	Human research participants
☒	☐	Clinical data

Methods

n/a	Involved in the study	
☒	☐	ChIP-seq
☐	☒	Flow cytometry
☒	☐	MRI-based neuroimaging

Antibodies

Antibodies used

Antibody | Source | Catalogue | RRID | Dilution
Nqo1 Abcam #ab34173 AB_2251526 1/1000
Nkx2.1/TTF-1 Dako #M3575 1/200
P63 Biocare Medical #CM163A AB_10582730 1/100
Hmga2 Biocheck #59170AP AB_2616589 1/1000
F4/80 Hybridoma N/A 1/100
CD68 Dako M0876 AB_2074844 1/200
Pro-SPC Merck AB3786 1/2000
CC10 Merck ABS1673 1/100
TALDO1 Sigma-Aldrich #HPA048089 AB_2680260 1/200
GAPDH Sigma-Aldrich G8795 AB_1078991 1/1000
EpCAM Biolegend #118217 AB_1501158 1/200
CD104 Biolegend #123603 AB_961034 1/200
CD45 Biolegend #103114 AB_312979 1/250
CD31 Biolegend #102417 AB_830756 1/250
CD3 Biolegend #100305 AB_312670 1/400
CD19 Biolegend #115511 AB_313646 1/400
CD4 Biolegend #100536 AB_493701 1/400
CD8 Biolegend #100708 AB_312747 1/400
FoxP3 Biolegend #320013 AB_439749 1/200
DX5/CD49b Biolegend #108909 AB_313416 1/200
NKp46/CD335 Biolegend #137603 AB_10552741 1/200
CD11c Biolegend #117309 AB_313778 1/200
CD11b Biolegend #101205 AB_312788 1/200
Ly6G Biolegend #127603 AB_1186105 1/400
CD103 Biolegend #121405 AB_535948 1/400

	Lyve1 Thermo Fisher PA5-22783 AB_11152044 1/200
Validation	All flow cytometry antibodies were first described in:<br>(1) Best et al., Synergy between the KEAP1/NRF2 and PI3K Pathways Drives Non-Small-Cell Lung Cancer with an Altered Immune Microenvironment, Cell Metabolism, 2018<br>Immunohistochemistry anibodies Nqo1, Nkx2.1, P-ERK, P-AKT, SPC, Keratin 8/18, CC10, Taldo1 were described previously in:<br>(1) Best et al., Synergy between the KEAP1/NRF2 and PI3K Pathways Drives Non-Small-Cell Lung Cancer with an Altered Immune Microenvironment, Cell Metabolism, 2018<br>F4/80 and CD68 were validated on normal mouse or human lung tissue and/or additional control tissues (mouse spleen, human tonsil) by immunohistochemical staining, to the satisfaction of S.A.B and K.D.S.

Eukaryotic cell lines

Policy information about cell lines

Cell line source(s)	All human cell lines (A549, H460, H441, H358) were obtained from ATCC.<br>Mouse primary cell lines were generated from tumor cells obtained from spontaneous lung tumors as indicated in the study.
Authentication	Quantitative RT-PCR was performed on all cell lines to confirm the identity based on presence/absence of KEAP1-loss/mutation.<br>In addition, Western blot analysis was performed on human cell lines to confirm identity.
Mycoplasma contamination	All cell lines were tested routinely for mycoplasma contamination and were routinely negative for infection.
Commonly misidentified lines<br>(See ICLAC register)	None.

Animals and other organisms

Policy information about studies involving animals; ARRIVE guidelines recommended for reporting animal research

Laboratory animals	Mouse strains used:<br>C57Bl6<br>KrasG12D/+<br>Keap1flox/flox<br>p53flox/flox<br>Lkb1flox/flox<br>All strains were on a pure 100% C57Bl6 background. Equal numbers of males and females were used in all experiments with age-matched littermate mice used as controls
Wild animals	The study did not involve wild animals
Field-collected samples	The study did not involve samples collected from the field
Ethics oversight	The Walter and Eliza Hall Institute Animal Ethics Committee

Note that full information on the approval of the study protocol must also be provided in the manuscript.

Human research participants

Policy information about studies involving human research participants

Population characteristics	For the KRAS-mutant cohort collected at St Vincent's Hospital by V.R. and G.W.<br>The study was blinded to all patient characteristics (age, gender, treatment, smoking status)
Recruitment	Patient samples (FFPE blocks) were recruited into the study based on (1) Lung adenocarcinoma diagnosis AND (2) positivity for KRAS-mutation. Mutation status of TP53 was identified for the study. Patient samples were obtained between 2013 to 2017, inclusive, at St Vincent's Hospital, Melbourne, Australia
Ethics oversight	The Walter and Eliza Hall Institute Human Research Ethics Committee

Note that full information on the approval of the study protocol must also be provided in the manuscript.

Flow Cytometry

Plots

Confirm that:

☒ The axis labels state the marker and fluorochrome used (e.g. CD4-FITC).

☒ The axis scales are clearly visible. Include numbers along axes only for bottom left plot of group (a 'group' is an analysis of identical markers).

☒ All plots are contour plots with outliers or pseudocolor plots.

☒ A numerical value for number of cells or percentage (with statistics) is provided.

Methodology

Sample preparation	Samples were prepared from the superior lobe of the mouse lung, strictly according to the methods chapter published by the authors S.A.B, A.K and K.D.S:<br>(1) Best et al., Combining Cell Type-Restricted Adenoviral Targeting with Immunostaining and Flow Cytometry to Identify Cells-of-Origin of Lung Cancer, Methods in Molecular Biology, 2018
Instrument	Flow cytometry analysis: BD LSR II<br>Flow cytometry sorting: BD FACSARIA III
Software	FlowJo LLC version 10 was used to analyse all flow cytometry data
Cell population abundance	Sorted tumor population was defined by CD45-EpCAM+ and was approximately 3-6 % of the total population. Macrophage sorted population was defined as CD45+CD11b+CD103- and was approximately 5-15 % of the total population.<br>Sorting efficiency was 80-100% as determined by the BD FACSARIA, using the P16Y16 sorting method into two tubes. Diluted cells and a low flow rate was always used to acquire sorted cells. Quantitative RT-PCR was used to define the purity of sorted populations.
Gating strategy	Tumor cell and macrophage gating can be found in the following citations:<br>(1) Best et al., Combining Cell Type-Restricted Adenoviral Targeting with Immunostaining and Flow Cytometry to Identify Cells-of-Origin of Lung Cancer, Methods in Molecular Biology, 2018<br>(2) Best et al., Synergy between the KEAP1/NRF2 and PI3K Pathways Drives Non-Small-Cell Lung Cancer with an Altered Immune Microenvironment, Cell Metabolism, 2018<br><br>Briefly, for cell sorting:<br>FSC-A/SSC-A: debris excluded<br>FSC-H/FSC-W: single cell gate<br>PI/FSC-A: live, PI negative cells gated<br>CD45-PEcy7/EpCAM-APCcy7: APCcy7+PEcy7-: EpCAM+ tumor cell population.<br>PEcy7+:<br>CD11b-APC/CD103-PE: APC+PE-: Alveolar macrophage population

☒ Tick this box to confirm that a figure exemplifying the gating strategy is provided in the Supplementary Information.
